# Pig-Derived Probiotic *Bacillus tequilensis* YB-2 Alleviates Intestinal Inflammation and Intestinal Barrier Damage in Colitis Mice by Suppressing the TLR4/NF-κB Signaling Pathway

**DOI:** 10.3390/ani14131989

**Published:** 2024-07-05

**Authors:** Heng Yin, Chengbi Wang, Yi Shuai, Zhuoya Xie, Jingbo Liu

**Affiliations:** School of Life Science and Engineering, Southwest University of Science and Technology, Mianyang 621010, China; yinheng@swust.edu.cn (H.Y.); chengbiwang10@163.com (C.W.); mynameisshuaiyi@163.com (Y.S.); xiezhuoya1232@163.com (Z.X.)

**Keywords:** *Bacillus tequilensis*, colitis, intestinal barriers, TLR4/NF-κB pathway

## Abstract

**Simple Summary:**

A new probiotic strain was screened from healthy adult pigs and named *B. tequilensis* YB-2. Through animal experiments, we found that adding *B. tequilensis* YB-2 to the diet can significantly reduce colitis induced by sodium dextran sulfate (DSS) and intestinal barrier damage in mice. The protective mechanism is probably realized by inhibiting the Toll-like receptor 4 (TLR4)/nuclear factor kappa-B (NF-κB) signaling pathway.

**Abstract:**

The search for new probiotics has been regarded as an important approach to improving intestinal health in animals. *Bacillus* has many advantages, such as strong resistance to harmful external factors, wide distribution, and easy colonization of the intestine. Hence, this study aims to screen for a probiotic *Bacillus* strain that improves animal intestinal health and to elucidate its probiotic mechanism so as to provide probiotic resources for the development of feed-using probiotic formulations. In this research, a strain of *Bacillus* was isolated from adult pig feces and named *B. tequilensis* YB-2. In vitro probiotic experiments showed that *B. tequilensis* YB-2 had strong acid and bile salt resistance, indicating that this strain can customize in the intestine. To further explore the effect of *B. tequilensis* YB-2 upon animal intestinal health, DSS-induced murine colitis models were established, and the body weight, colonic morphology, inflammatory cytokines level, and intestinal-barrier- and TLR4/NF-κB-pathway-related protein were determined. The results showed that mice receiving drinking water with 3% DSS were found to develop colitis symptoms, including body weight loss and increased disease activity index (DAI); colon length and microvilli shedding were shortened; tight junctions were disrupted; goblet cells decreased; anti-inflammatory cytokines were inhibited; and pro-inflammatory cytokines and the TLR4/NF-κB signaling pathway were activated. Notably, orally received *B. tequilensis* YB-2 alleviated symptoms of DSS-induced colitis in mice. The above results indicated that *B. tequilensis* YB-2 was capable of improving colitis in mice by weakening inflammation and intestinal barrier damage, and its mechanism may involve the TLR4/NF-κB pathway. Overall, this research suggests that *B. tequilensis* YB-2 has the potential to serve as an animal feed additive to prevent intestinal inflammation.

## 1. Introduction

Antibiotics, widely used as early feed additives in the livestock and poultry industry, play a significant role in the prevention and treatment of animal diseases and the promotion of animal growth. However, with the widespread and even excessive use of antibiotics in animal feed, their adverse effects are becoming increasingly prominent, leading to the development of antibiotic resistance in pathogens, imbalances in the microbial flora within animals, and a decline in immune function. This directly endangers human health and the ecological environment through the presence of drug residues in animal products [1,2]. Given this, searching for alternatives to antibiotics has become a hot topic around the world. Probiotics are live microbial supplements that have been highly regarded due to their non-toxic and residue-free characteristics [3]. Apart from the wide application in traditional food and health industries, probiotics have also shown significant effects in improving intestinal flora balance, promoting the growth of beneficial bacteria, enhancing immunity, and so on. Therefore, they can be used as alternatives to antibiotics and harmful chemicals [4]. 

Currently, the most-studied probiotics are *Lactobacillus*, *Bifidobacterium*, and *Streptococcus* (non-spore-forming bacteria), but they are mostly sensitive to stomach acid and other unfavorable environments, and their activity is easily damaged. Compared to non-spore bacteria, *Bacillus* can produce endospores and has excellent tolerance to high temperature and acidity. It can maintain good activity during processing and storage and can produce various antibacterial materials to suppress the growth of pathogenic microorganisms [5]. Therefore, *Bacillus* is highly regarded as a probiotic. Currently, *Bacillus* strains, including *B. clausii*, *B. subtilis*, *B. coagulans*, *B. polyfermentans*, *B. cereus*, and *B. licheniformis*, have been used commercially as dietary supplements, growth stimulants, and competitive exclusion agents for humans and animals [6]. Numerous studies have confirmed that adding *B. subtilis* preparations to animal feed can enhance the immune functions of livestock and poultry. Additionally, it can also improve the feed digestibility and the hygiene of the feeding environment, which is beneficial to the healthy development of the livestock and poultry farming industry [7,8]. 

The intestine is not only an important organ in the digestion of food; it is also an important immune organ in the body. The structure of intestinal barriers, consisting of the mucus layer and intestinal epithelium, can resist the invasion of most harmful substances. When the intestinal barrier is damaged, the intestinal permeability increases, which allows pathogens to enter the body and causes intestinal inflammation. This represents the main pathogenic mechanism of most intestinal diseases [9]. The most common inflammatory bowel diseases (IBDs) include ulcerative colitis (UC) and Crohn’s disease (CD) [10]. In the livestock industry, factors such as rough feed, improper mixing, mold contamination, improper use of drugs, and the overuse of antibiotics can all cause intestinal inflammation in animals. With the occurrence of intestinal inflammation, the excessive production of pro-inflammatory cytokines and inflammatory mediators may result in intestinal digestive and absorptive dysfunctions, thereby damaging the nutrient transport and absorption throughout the intestine and reducing the growth performance of animals. Currently, researchers have found that probiotics can downregulate the expression levels of inflammatory markers in the intestines, achieve an anti-inflammatory effect, and play an important role in maintaining intestinal health [11,12]. 

Antibiotics used to be the main effective ingredient for preventing and treating intestinal inflammation in animals, but due to the various drawbacks caused by the overuse of antibiotics, many countries have now completely banned the application of antibiotics to animal feed. Because probiotics are one of the safe, effective, environmentally friendly, and green alternatives to antibiotics due to their good characteristics, this study aims to isolate and identify probiotic *Bacillus* from adult healthy pigs and study its biological characteristics. Meanwhile, using colitis mice induced by dextran sodium sulfate (DSS) as experimental models, this study probed into the effects of porcine-derived *Bacillus* upon intestinal inflammation, explored its probiotic effects and possible mechanisms, and provided theoretical guidance and technical support for the application of porcine-derived probiotic *Bacillus* to animals, as well as technical support for antibiotic alternatives.

## 2. Materials and Methods

### 2.1. Isolation and Identification of Pig-Derived Bacillus

Feces were taken from a local pig farm in Mianyang, transported in ice packs, and then stored in Southwest University of Science and Technology. 

After being placed in saline buffer at 100 °C for 15 min, 0.1 mL of pig feces were aspirated onto LB agar plates and incubated for 24 h at 37 °C, followed by purification of different morphological colonies and checking for Gram and spore staining. Then, 16S rRNA genes were sequenced to further identify the resultant isolates. Finally, MEGA 7.0 software was used to create an evolutionary tree according to BLAST comparison results. Named *B. tequilensis* YB-2, the identified isolates were preserved in China Center for Type Culture Collection (CCTCC No. M 20221531). 

### 2.2. Growth Characteristics and Acid and Bile Salts Tolerance of B. tequilensis YB-2

After being inoculated into LB broth (2% *v*/*v*), *B. tequilensis* YB-2 was put in a shaker to be centrifuged at 180 rpm/min (37 °C) for 24 h. Then, the OD600 nm value was tested every 2 h, with 3 reads in each session. 

Tolerance to low pH and bile salt was assessed using sterile phosphate-buffered saline (PBS) with different pH values (2. 0 and 4. 0) or using sterile PBS (Solarbio, Beijing, China) containing different bile salt (Solarbio, Beijing, China) levels (0.15%, and 0.3%) to incubate 1% activated *B. tequilensis* YB-2. After incubating the mixture at 37 °C for 2 h to 4 h, the OD600 nm value was recorded. Lastly, the following formula was utilized to calculate the survival rate:

$$Survival\;\left(\%\right)=\frac{Experimental\;groupOD\;-\;Blank\;groupOD}{Control\;groupOD\;-\;Blank\;groupOD}\times 100\%$$

### 2.3. Antibacterial Activities of B. tequilensis YB-2

Antimicrobial activities of *B. tequilensis* YB-2 were evaluated against three associated pathogenic bacteria: *Escherichia coli* (*E. coli* O157:H7), *Staphylococcus aureus* (*S. aureus* ATCC6538), and *Salmonella enteritidis* (*S. enteritidis* CMCC50335). All strains were attained from the Laboratory of Microbiology, Sichuan Agricultural University, Chengdu City, Sichuan Province. After spreading pathogenic bacteria uniformly on the LB agar plates with sterile cotton swabs, the plates were perforated. An antibiotic (doxycycline hydrochloride), *B. tequilensis* YB-2 solution, or normal saline were separately added in each hole of the medium to co-culture with various pathogens in an incubator at 37 °C for 24 h. This was followed by measurement of the diameter of the inhibition zone. Three repetitions were set for each group. 

### 2.4. Animals and Study Design

Seventy-two male ICR mice of 4 weeks, SPF grade, weighing 18–22 g, were provided by Chengdu DOSSY experimental animals Co., LTD (Chengdu, China). All mice were kept in separated cages and raised at 25 ± 2 °C with a 12 h light–dark cycle under a standard SPF environment. The mice had free access to water and food. All experimental procedures followed the guidelines of the Animal Care and the Ethics Committee, Southwest University of Science and Technology, Mianyang, China (approval No: L20230453). These 72 mice were randomly classified into four groups (18 mice/group): a control group, a DSS group, a YB-2 group, and a DSS + YB-2 group. During the four-week trial, mice in the control group and DSS group orally received intragastric administration of sterile saline (100 μL per mouse) daily, while corresponding bacterial suspension (1×10^10^ CFU/mL, 100 μL per mouse) was provided to mice in the YB-2 group and DSS + YB-2 group every day. After four weeks of intragastric administration, the drinking water of the mice in the DSS group and DSS+ YB-2 group was supplemented with 3% (*w*/*v*) DSS (molecular weight = 5 kDa, Shanghai Yuanye Bio-Technology Co., Ltd. Shanghai, China) for 7 days to induce acute colitis (Table 1). The establishment of a colitis mouse model in this research followed previous studies [13]. Afterward, the mice were sacrificed to carry out the subsequent experiments. 

### 2.5. Assessment of Disease Activity Index

The method proposed by Hossen et al. [13] and Liu et al. [14] was used to determine the disease activity index (DAI) score. The DAI score mainly involves three components: weight change, stool characteristics, and blood in stool. The following specific scoring criteria was adopted: body weight loss (0: none, 1: 1–5%; 2: 5–10%; 3: 10–15%; 4: >15%); fecal occult blood [0: no bleeding; 1: occult blood (negative); 2: occult blood (positive); 3: bleeding; 4: gross bleeding]; stool trait [0: normal; 1: loose stools (forming); 2: loose stool (no forming); 3: diarrhea (slight); 4: diarrhea (serious)]. Finally, the following formula was used to calculate the DAI score:

$$The\;DAI\;score=\frac{weight\;change\;score\;+\;fecal\;blood\;score\;+\;stool\;trait\;score}{3}$$

### 2.6. Histological Evaluation of Colon Tissue

After euthanizing the mice, the researchers dissected and photographed the colons and measured the colon length. This was followed by fixing colon samples in 4% paraformaldehyde, ethanol dehydration, paraffin embedment, slicing the fixed colon tissues into sections of 5 μm thick, and hematoxylin and eosin (H. E.) staining. Tissue pathological changes were observed and imaged using a digital microscope camera (Leica DM500, Wetzlar, Germany).

The histopathological score followed previous studies with slight modifications [15,16,17]. Each colon was evaluated histologically blinded, and the evaluation parameters included inflammatory infiltration (3: severe; 2: moderate; 1: mild; 0: none), intestinal epithelial cell loss (3: severe; 2: moderate; 1: mild; 0: none), damage to crypts (3: intact epidermis only; 2: damaged basal 2/3 of crypts; 1: destroyed bottom 1/3 of crypts; 0: none;), and decrease in intestinal mucosal thickness (3: severe; 2: moderate; 1: mild; 0: none). A total histological severity score, ranging from 0 to 12, was obtained by summing the four item scores.

### 2.7. Transmission Electron Microscope Observation

After dissecting the mice and cutting the colon into small pieces, the sliced colon was fixed by being placed into 2.5% glutaraldehyde immediately and post-fixed in 2% veronal acetate-buffered OsO_4_. The sample tissues dehydrated through gradient acetone were embedded in epoxy resin. A glass knife was utilized to slice sample blocks into sections (65–75 nm) in a microtome, which were then placed in copper grids with no coatings. After using lead citrate and uranyl acetate to stain the tissue sections, a transmission electron microscope (Hitachi, H-600 transmission, Tokyo, Japan) was used to observe the ultrastructural architectures of the colon.

### 2.8. Alcian Blue/Periodic Acid-Schiff (PAS) Staining

After being de-waxed, stained in 1% Alcian blue for 5 min, oxidized in 1% periodic acid, and immersed in Schiff’s reagent, the colon sections were mounted and observed under a light microscope. The section was rinsed with water in each step. The stained goblet cells were blue.

The counting method of goblet cells was that of Wang et al. [18]. Five sections were taken for each tissue, and five images (400×) were taken randomly for each section. Then, the goblet cells were counted by using the Image Pro Plus v6.0 software (Media Cybernetics Inc., Bethesda, MD, USA), and the selection principle for goblet cells was to select the one with more secretion and an intact section. The area of each image is 0.064 mm^2^, so the count of goblet cells was expressed as numbers/0.064 mm^2^.

### 2.9. Detection of Nitric Oxide in the Serum and Colon

Blood samples were collected from orbital veins of anesthetized mice by the end of the experiments. After centrifuging the samples at 3000 rpm for 10–15 min, serum was obtained for later detection. Ice-cold saline was used to dilute the colon tissues, which were then homogenized through use of a homogenizer to obtain homogenates. The resultant homogenates were centrifuged at 3000 rpm for 10–15 min, thus acquiring the supernatants. The Beyotime (Beyotime Biotechnology Co., Ltd., Shanghai, China) kits were utilized to measure nitric oxide (NO) levels in serum and colon homogenate.

### 2.10. Serum Cytokine Concentration

The serum samples were prepared in the same way as in step 2.9. Specific ELISA kits produced by Biosharp (Hefei, China) were used to measure the interleukin (IL)-1β, IL-6, tumor necrosis factor α (TNFα), IL-4, and IL-10 concentrations in serum following the manufacturer’s instructions.

### 2.11. Quantitative Real-Time PCR (qRT-PCR)

After being collected and ground with liquid nitrogen using a pestle and mortar, the colon tissues were preserved at −80 °C. Renal total RNA was extracted using RNAiso Plus (9108/9109, Takara, Otsu, Japan) according to the manufacturer’s recommendations. This was followed by synthesizing complementary DNA (cDNA) using the Prim-ScriptTM RT reagent kit (RR047A, Takara, Japan) following the manufacturer’s instructions. Then, qRT-PCR was performed with the SYBR Premix Ex TaqTM II kit (DRR820A, Takara, Japan) on LightCycler^®^ 480 Real-Time PCR System (Roche, Basel, Switzerland). Table 2 lists the qPCR primers. The gene expression was normalized using β-actin. The 2^−ΔΔCt^ method was used to calculate the relative mRNA expression levels of interleukin (IL)-6, IL-1β, IL-10, IL-4, TNFα, MUC2 (mucoprotein 2), ZO-1 (zonula occludens-1), occludin, claudin-1, TLR4 (Toll-like receptor 4), and NF-κB (nuclear factor kappa-B). 

### 2.12. Western Blotting

RIPA lysis buffer was used to extract the protein in colon samples, the concentration of which was measured using BCA protein assay reagent (P0010S, Beyotime Technology, Shanghai, China). After SDS-PAGE, protein samples were transferred to nitrocellulose filter membranes blocked with 5% skimmed milk for 1 h at the room temperature. Then, the protein samples were incubated with the primary antibody overnight at 4 °C. The primary antibodies were as follows: MUC2 (1:1000, A4767, Abclone, Wuhan, China), ZO-1 (1:500, A0659, Abclone, Wuhan, China), occludin (1:1000, A2601, Abclone, Wuhan, China), claudin-1 (1:1000, A11530, Abclone, Wuhan, China), TLR4 (1:1000, A11226, Abclone, Wuhan, China), NF-κB (1:1000, 8242s, CST, Beverly, MA, USA), p-NF-κB (1:1000, 3033s, CST, Beverly, MA, USA), and β-actin (1:1000, AC026, Abclone, Wuhan, China). After incubation with horseradish-peroxidase-conjugated secondary antibody (diluted 1:3000) for 1 h, a Chemidoc XRS was adopted to detect protein bands on the membranes by utilizing the ECL kit (P0018A, Beyotime Technology, Shanghai, China). Quantity analyses were performed to show relative protein expressions to β-actin.

### 2.13. Statistical Analysis

The PROC UNIVARIATE procedure of SAS 9.2 (SAS Inst. Inc., Cary, NC, USA) was employed for the purposes of verifying the normality and homogeneity of the variance of the variables. The data were analyzed using analysis of variance (ANOVA) with a statistical model including DSS, YB-2, and their interaction. The interaction was significant; thus, Tukey’ test was used to compare the simple means. The results were expressed as the mean ± standard error of the mean (SEM). A value of *p* < 0.05 was deemed to be statistically significant. The *p*-values for each comparison were shown in Tables S1–S5. The results were subsequently plotted by using GraphPad Prism 8 software.

## 3. Results

### 3.1. Identification of B. tequilensis YB-2

**Figure 1:**
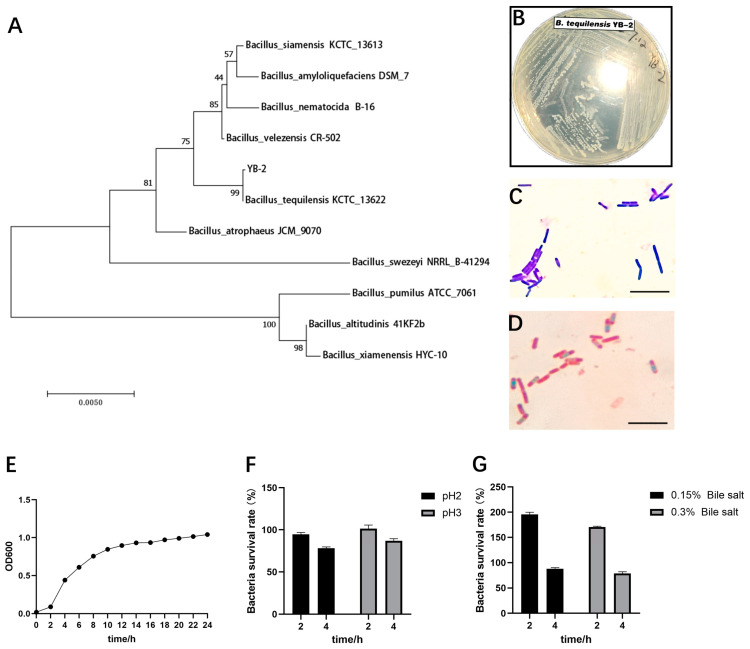
Identification and probiotic properties of *B. tequilensis* YB-2: (**A**) the phylogenetic tree of *B. tequilensis* YB-2; (**B**) colony morphology of *B. tequilensis* YB-2; (**C**) Gram staining of *B. tequilensis* YB-2, bar = 10 μm; (**D**) spore staining of *B. tequilensis* YB-2, bar = 10 μm; (**E**) the growth curve of *B. tequilensis* YB-2; (**F**) the ability of acid tolerance of *B. tequilensis* YB-2; (**G**) the ability of bile salt tolerance of *B. tequilensis* YB-2.

Figure 1B displays colonial morphologies. The colonies had a diameter of about 1–2 mm, were milky white in color, were opaque, had a smooth surface, and were partly raised. This proved that the *B. tequilensis* YB-2 strains were Gram-positive bacteria, and the spore stain was positive (Figure 1C,D). A total of 16S rDNA sequencing results were submitted to the NCBI database to be detected by using the basic local alignment search rool (BLAST), and the consanguinity was analyzed by use of the phylogenetic tree. The results showed that the strains were highly (99%) homologous with *B. tequilensis* (Figure 1A). Therefore, the strain was named *B. tequilensis* YB-2.

### 3.2. Probiotic Properties of B. tequilensis YB-2 In Vitro

By measuring its growth curve, *B. tequilensis* YB-2 was found to enter the logarithmic growth phase and stable phase separately after 2 and 10 h (Figure 1E). Meanwhile, *B. tequilensis* YB-2 was highly tolerant to acids at pH 2 and pH 3, with separate survival rates of 78.25% and 86. 99% for 4 h (Figure 1F). In addition, *B. tequilensis* YB-2 also had strong bile salt tolerance as evinced by its separate survival rates of 88.01% and 78.75% in 0.15% and 0.3% bile salts (Figure 1G). The above results support that *B. tequilensis* YB-2 colonized and survived in the gut.

### 3.3. Antibacterial Activity of B. tequilensis YB-2

**Figure 2:**
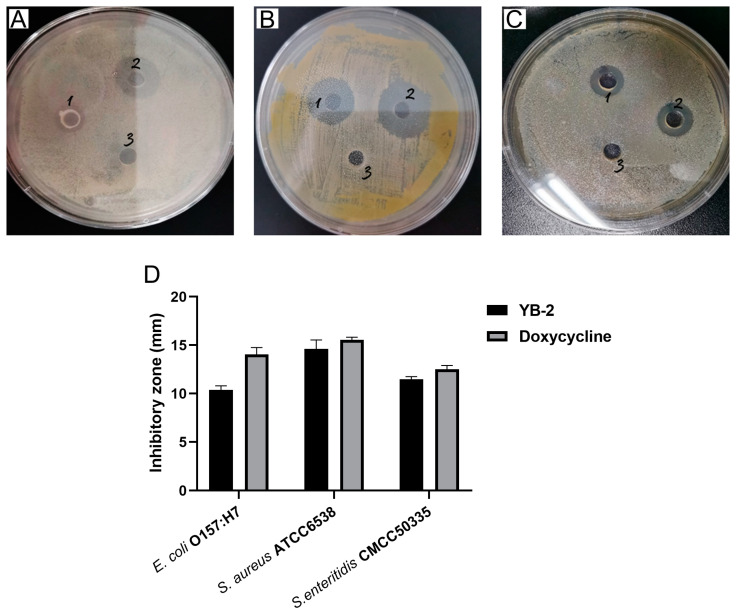
Antibacterial activity of *B. tequilensis* YB-2: (**A**–**C**) the inhibitory effect of *B. tequilensis* YB-2 against *E. coli* O157:H7 (**A**), *S. aureus* ATCC6538 (**B**), and *S.enteritidis* CMCC50335 (**C**) (hole 1 is the group of *B. tequilensis* YB-2; hole 2 is the positive control group (doxycycline); hole 3 is the negative control group); (**D**) the diameter of the inhibition zone of *B. tequilensis* YB-2 against pathogenic bacteria.

The detection results of antimicrobial activities suggested the inhibitory effects of *B. tequilensis* YB-2 on the growth of *E. coli* O157:H7, *S. aureus* ATCC6538, and *S. enteritidis* CMCC50335. Moreover, it had a stronger inhibitory effect on *S. aureus* ATCC6538 compared with others (Figure 2A–D).

### 3.4. B. tequilensis YB-2 Alleviates Weight Loss and Lowers DAI Scores

**Figure 3:**
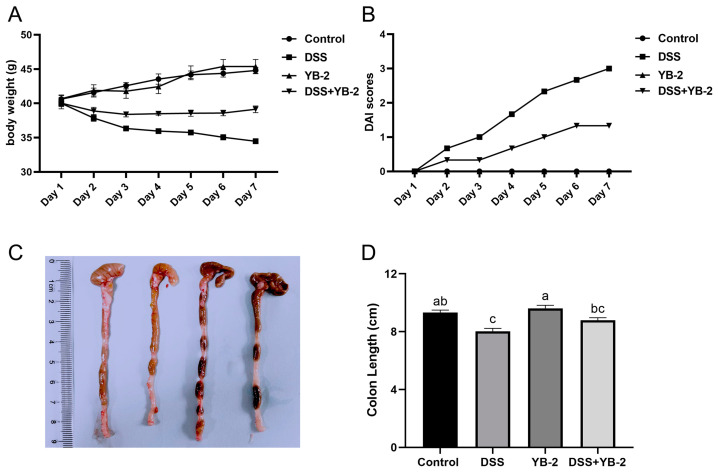
Effect of *B. tequilensis* YB-2 on the weight and colon length in colitis mice model: (**A**) changes in body weight; (**B**) disease activity index scores; (**C**,**D**) colon length. Values are presented with the means ± SEM (n = 6). Significant difference was expressed with different letters among different groups at *p* < 0.05.

It can be seen from Figure 3A that mice in the DSS group and the DSS + YB-2 group began to lose body weight on day two, with a higher weight loss rate in the DSS group than in the DSS + YB-2 group. On the contrary, the body weights of mice in the control group and the YB-2 group increased from day one to day seven. The DAI scores suggest the severity of colitis (Figure 3B). The DAI scores of mice in the DSS group and the DSS + YB-2 group started to rise and surpass that of those in the control group on day two. Additionally, the DAI score of mice in the DSS + YB-2 group showed a gradual increasing trend, which is remarkedly slower than that of the DSS group. These findings indicated that *B. tequilensis* YB-2 was capable of lessening weight loss and diarrhea in DSS-induced colitis mice.

### 3.5. B. tequilensis YB-2 Alleviates Colon Damage

To examine whether *B. tequilensis* YB-2 is able to alleviate colon tissue damage or not, the colon length of mice was measured, and tissues were stained and observed under a transmission electron microscope (TEM). The results demonstrated that the colon length of mice was significantly shorter in the DSS group than in the control group (*p* < 0.05) (Figure 3C,D). Mice in the DSS + YB-2 group were found to have a significantly longer colon length than those in the DSS group (*p* < 0.05), indicative of the ability of *B. tequilensis* YB-2 to reduce colon shortening in colitis mice induced by DSS.

**Figure 4:**
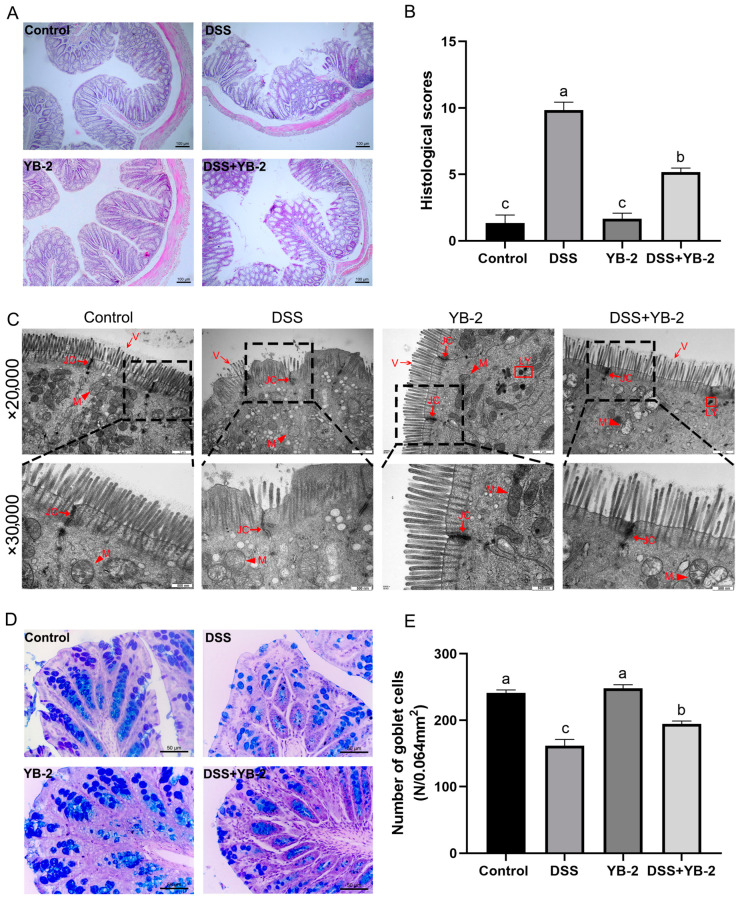
Effect of *B. tequilensis* YB-2 on colonic histology in colitis mice model: (**A**) H.E. staining of the colonic tissue, ×100, bar = 100 μm; (**B**) histological scores; (**C**) the ultrastructural structure of the colonic tissue, ×20,000 or ×30,000, bar = 1 μm or 500 nm (M: mitochondria (▲); V: microvilli (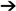); LY: lysosomes (**□**); JC: junctional complexes (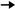)); (**D**) Alcian blue/PAS staining of colonic tissue, ×400, bar = 50 μm; (**E**) the number of goblet cells in colon. Values are presented with the means ± SEM (n = 6). Significant difference was expressed with different letters among different groups at *p* < 0.05.

H.E. staining results of the colon are illustrated in Figure 4A. Inflammatory cell infiltration is found in colon tissues in the lamina propria of mice in the DSS group, which also exhibit detached partial epithelial at the apical of the mucosa, a thinned mucosal layer, and reduced glands. On the contrary, the colon tissues of mice in the DSS + YB-2 group are minorly damaged, along with slight inflammatory cell infiltration, slight mucosal epithelial detachment, and a largely intact mucosa. The control group and the YB-2 group do not show obvious pathological damage to the colon.

The TEM images of colon tissues are shown in Figure 4B. In the DSS group, some microvilli of colon absorption cells were reduced, shortened, or even completely shed, the cell junction gap was slightly widened, intracytoplasmic vacuoles were found, mitochondria were reduced and swollen, and the structure was unclear. Additionally, although the microvilli and cell connections in the DSS + YB-2 group were relatively normal, the mitochondria were still swollen, and the structure was unclear. Also, no obvious ultrastructure change was observed in the colon in the control group and YB-2 group.

The Alcian blue/PAS staining results are shown in Figure 4C. Stained goblet cells in mucosal epithelia in the colon are blue or purple. The number of goblet cells was assessed in Figure 4D. Compared with the control group, the DSS group had a significantly smaller number of goblet cells in the colon (*p* < 0.05). The DSS + YB-2 group had a significantly higher number of goblet cells compared with the DSS group (*p* < 0.05). The number of goblet cells do not differ significantly between the control and YB-2 groups (*p* > 0.05).

### 3.6. B. tequilensis YB-2 Decreases Expression of Inflammatory Cytokines

**Figure 5:**
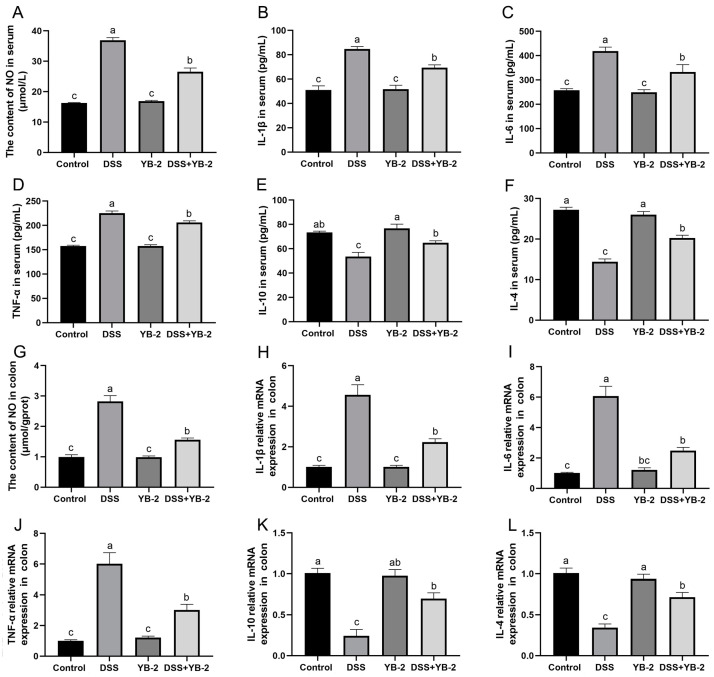
Effect of *B. tequilensis* YB-2 on inflammation in colitis mice model: (**A**) the content of NO in serum; (**B**–**F**) the levels of IL-1β, IL-6, TNFα, IL-10, and IL-4 in serum; (**G**) the content of NO in the colon; (**H**–**L**) the mRNA expression of IL-1β, IL-6, TNFα, IL-10, and IL-4 in the colon. Values are presented with the means ± SEM (n = 6). Significant difference was expressed with different letters among different groups at *p* < 0.05.

NO is considered as an inflammatory mediator during IBDs [19]. DSS treatment resulted in a significant increase in the NO levels of serum and the colon (Figure 5A,G) in comparison with the control group (*p* < 0.05), and the DSS + YB-2 group had a significantly higher NO level than the DSS group (*p* < 0.05).

The levels of pro-inflammatory cytokines have a correlation with inflammation severity. The DSS group showed much higher serum levels of pro-inflammatory cytokines (IL-1β, IL-6, and TNFα) than the control group (*p* < 0.05). Different from this, the DSS + YB-2 group exhibited significantly lower levels of pro-inflammatory cytokines (*p* < 0.05) compared to the DSS group (Figure 5B–D). In addition, the mRNA expression levels of these pro-inflammatory cytokines in colon tissues were detected by conducting qRT-PCR. The DSS + YB-2 group displayed significantly lower mRNA expression levels of IL-6, IL-1β, and TNFα than the DSS group (*p* < 0.05), and the DSS + YB-2 group and the control group displayed no significant difference in these cytokines (*p* > 0.05) (Figure 5H–J).

Furthermore, the levels of anti-inflammatory cytokines (like IL-4 and IL-10) were also detected in our study. The IL-4 and IL-10 had significantly lower levels in the DSS group (*p* < 0.05) than in the control group. However, treatment with *B. tequilensis* YB-2 had a significant effect on reducing the DSS-induced serum levels of IL-10 and IL-4 (*p* < 0.05) (Figure 5E,F). Similarly, the mRNA levels of these anti-inflammatory cytokines in colon tissues showed a consistent trend with the results observed in serum. The DSS + YB-2 group displayed significantly higher mRNA expression levels of IL-10 and IL-4 than the DSS group (*p* < 0.05), and the DSS + YB-2 group and control group do not differ remarkably in these cytokines (*p* > 0.05) (Figure 5K,L).

The above results point out that *B. tequilensis* YB-2 was able to alleviate colitis induced by DSS through regulation of the levels of inflammatory cytokines.

### 3.7. B. tequilensis YB-2 Repairs the Intestinal Mucosal Barrier

**Figure 6:**
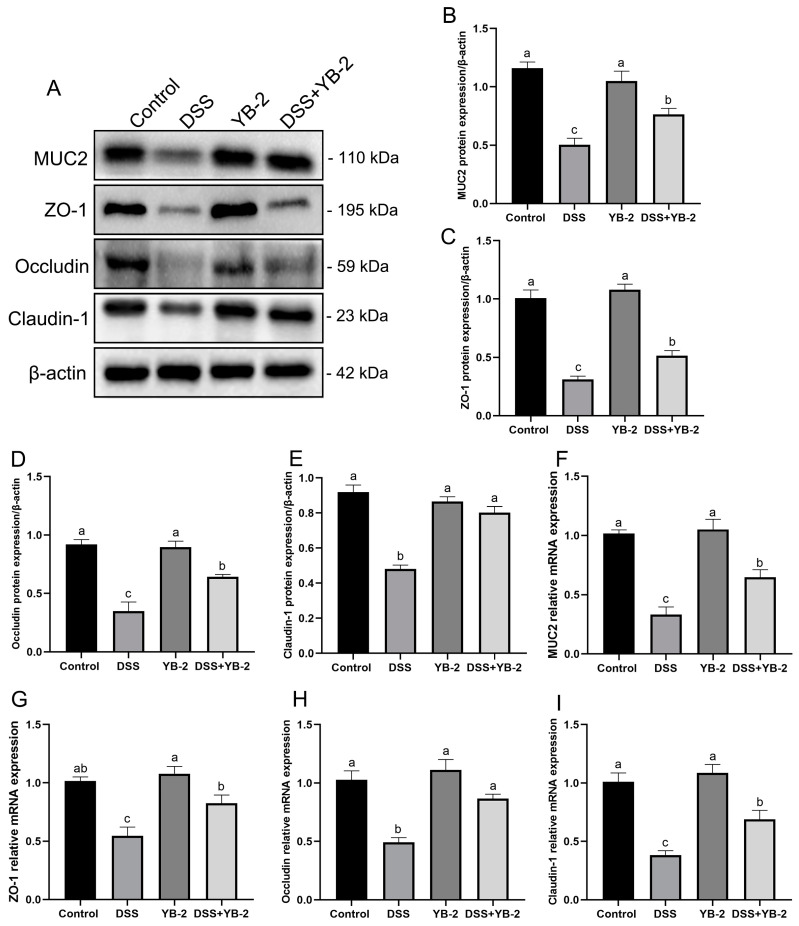
Effect of *B. tequilensis* YB-2 on the expression of genes related to tight junctions and mucins in the colon: (**A**) the Western blot results of MUC2, ZO-1, occludin, and claudin-1 protein expression; (**B**–**E**) the quantification of MUC2, ZO-1, occludin, and claudin-1 protein expression; (**F**–**I**) the mRNA expression of MUC2, ZO-1, occludin, and claudin-1. Values are presented with the means ± SEM (n = 6). Significant difference was expressed with different letters among different groups at *p* < 0.05.

MUC2 is the skeleton of colonic mucus constituting the physical intestinal barrier [20]. Figure 6A,B,F show that the DSS group had significantly lower mRNA and protein expression of MUC2 than the control group. However, the DSS + YB-2 group exhibited higher mRNA and protein expression of MUC2 than the DSS group.

In addition, occludin, ZO-1, and claudin-1 are proteins, which were essential for forming tight junctions (TJs) between colonic mucosal cells. Compared to the control group, the DSS group showed significantly lower protein and mRNA expression levels of ZO-1, occludin, and claudin-1 (*p* < 0.05) (Figure 6A,C–E,H,I). However, compared with the DSS group, the DSS + YB-2 group had higher mRNA and protein expression levels of ZO-1, occludin, and claudin-1.

These findings indicate that *B. tequilensis* YB-2 had repair and protection effects on the intestinal mucosal barrier (IMB).

### 3.8. Effect of B. tequilensis YB-2 on the TLR4/NF-κB Signaling Pathway of Colitis Mice

**Figure 7:**
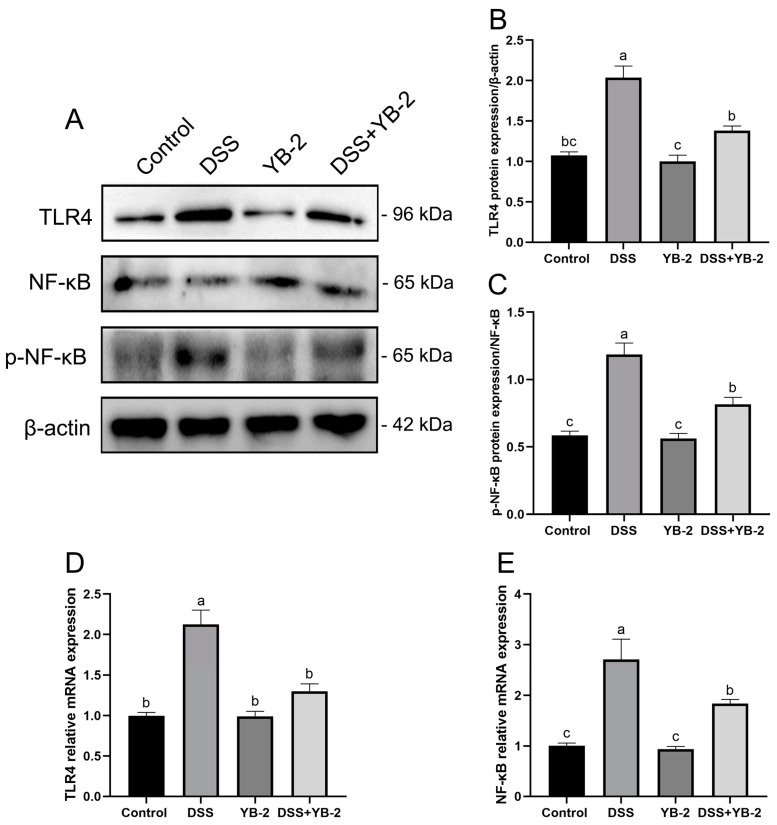
Effect of *B. tequilensis* YB-2 on the TLR4/NF-κB signaling pathway in the colon: (**A**) the Western blot results of TLR4, NF-κB, and p-NF-κB protein expression; (**B**) the quantification of TLR4 protein expression; (**C**) the quantification of p-NF-κB/NF-κB protein expression; (**D**) the mRNA expression of TLR4; (**E**) the mRNA expression of NF-κB. Values are presented with the means ± SEM (n = 6). The columns marked with different letters are significant differences (*p* < 0.05).

For further investigating the molecular mechanism by which *B. tequilensis* YB-2 alleviates intestinal inflammation and intestinal barrier destruction, the TLR4/NF-κB signaling pathway was examined in the colon. Compared to the control group, the DSS group was found to have significantly higher protein expression levels of TLR4 and p-NF-κB (*p* < 0.05). However, the DSS + YB-2 group showed lower protein expression levels (*p* < 0.05) than the DSS group (Figure 7A–C).

Figure 7D,E display mRNA expression levels of NF-κB and TLR4. The results show that the influence of *B. tequilensis* YB-2 on the mRNA expression levels of NF-κB and TLR4 of hearts was similar to that on the protein expression. The DSS + YB-2 group showed mRNA expression levels of NF-κB and TLR4 in the colon which were significantly decreased compared to the DSS group. In addition, the control group and YB-2 group had no statistically significant difference in terms of mRNA expressions of TLR4 and NF-κB.

## 4. Discussion

*Lactobacillus* and *Bifidobacteria*, which are inherent bacteria in probiotics for animal feed, have high nutritional requirements for the gastrointestinal tract and strict growth conditions, making them unsuitable for industrial-scale production. *Bacillus* needs low nutritional requirements and simple cultivation conditions. As a probiotic, it can improve the digestibility and nutritional value of feed and has strong resistance to harsh conditions such as gastric acid, bile, and radiation [21,22]. The obtained spore dormancy has good stability and can germinate into nutritional cells in the intestine to participate in the competition for nutrients and living space with harmful bacteria in the intestinal flora and can stimulate the immune system to improve host function. Therefore, the purpose of this research is to isolate probiotic *Bacillus* strains from locally raised antibiotic-free healthy adult pigs. After identification by 16S rDNA sequencing, we named this strain *B. tequilensis* YB-2. By testing probiotic characteristics, we found that *B. tequilensis* YB-2 had certain acid and bile salt tolerances and a strong inhibitory effect on pathogenic bacteria, including *E. coli* O157:H7, *S. aureus* ATCC6538, and *S. enteritidis* CMCC50335. These results indicated that *B. tequilensis* YB-2 was highly tolerant to the intestinal environment, that it exerted an antibacterial effect, and that it had the potential to serve as a substitute probiotic. Similarly, Hong-Jin et al. [23] found that an isolated strain of *B. tequilensis* JBC17126 from traditional soybean paste also had good acid and bile salt tolerances and exhibited good inhibitory effects against *B. cereus* and *S. aureus*, which made it a potentially useful probiotic. In addition, Zahoor et al. [24] also found that the enzyme produced by the screened *B. tequilensis* ZMS-*2* fermentation had antibacterial activities against *Klebsiella pneumoniae*, *S. aureus*, and *E. coli*.

To further validate the potential application of *B. tequilensis* YB-2 in animals, a DSS-induced colitis model was established to explore the efficacy and possible mechanism of *B. tequilensis* YB-2 on colitis in mice. DSS is one of the most widely used and important chemical substances for inducing IBDs. Treating mice with drinking water containing DSS leads to severe colitis, which is characterized by body weight loss, diarrhea, bloody stools, and even death [25]. In this study, we first pre-treated mice with *B. tequilensis* YB-2 for four weeks and then induced acute colitis in mice by adding 3% DSS to drinking water. We found that the weight loss trend of colitis mice treated with YB-2 pre-processing decreased, and the changes in fecal characteristics and intestinal bleeding were better than those of colitis mice treated with DSS. At the same time, the shortening of intestinal length caused by colitis was significantly alleviated in mice of the YB-2 + DSS group. In addition, it was found through histopathological observation that YB-2 pretreatment significantly alleviated the pathological damage caused by DSS, including increased colonic mucosal hyperplasia, inflammatory cell infiltration, epithelial cell shedding, villus loss, disruption of TJs between epithelial cells, and decreased goblet cells. Therefore, the results indicated that oral pretreatment with *B. tequilensis* YB-2 can significantly alleviate the severity of colitis in mice and that it had a preventive effect. This conforms to the results of intestinal inflammation damage caused by other *Bacillus* strains. Jia et al. [26] found that potential probiotic *B. licheniformis* ZW3 isolated from camel feces could reduce the body weight, colon shortening, and DAI of colitis mice. Li et al. [27] also revealed that administration of *B. licheniformis* can alleviate various colitis symptoms induced by DSS, such as weight loss, DAI increase, and disruption of intestinal barrier integrity.

As a signaling molecule that can regulate cell function, NO has involvement in colitis development and even in carcinogenesis [28]. In the early stage of enteritis, a small amount of NO can protect the epithelium and plays an anti-inflammatory role; whereas, as the inflammation degree increases, a large amount of NO will cause oxidative damage to the epithelium, thereby exacerbating the inflammatory response [28,29]. The cytokines TNF-α, IL-1β, and IL-6 represent critical pro-inflammatory substances of enteritis [30]. IL-10 and IL-4 are common anti-inflammatory cytokines, playing a crucial part in mediating humoral immune response, infectious disease, etc. [31,32]. This study found that compared with colitis models induced by DSS, expression levels of NO, TNF-α, IL-1β, and IL-6 were remarkably reduced in the peripheral blood and colon tissues of colitis mice pre-treated by *B. tequilensis* YB-2, while expression levels of IL-10 and IL-4 significantly increased. This indicates that *B. tequilensis* YB-2 has a certain preventive and therapeutic effect on enteritis. Other *Bacillus* species have also been reported to present similar research findings. Zhang et al. [33] stated that adding *B. subtilis* to the diet of broiler chickens can reduce TNF-α, IL-6, IL-1β, and IL-18 levels in serum and jejunal mucosa and can also alleviate intestinal inflammation. Hee-Su et al. [34] also demonstrated that *B. subtilis* P223, which has probiotic properties, can decrease NO production and downregulate the expression levels of pro-inflammatory cytokines IL-6, TNF-α, and IL-1β in the RAW 264. 7 inflammation model induced by LPS.

IMB, composed of different epithelial cells connected by TJs and host-secreted mucus layers, serves as the first line of defense in preventing pathogen invasion. TJs, as the most common intercellular connection, formed by proteins including ZO-1, occludin, and claudins, are core components of the intestinal barrier [35,36]. Multiple studies have shown that colitis patients altered TJ structure in intestinal epithelium, with significant downregulation of TJ proteins occludin and ZO-1, causing the increase in intestinal permeability [37,38]. Colitis induced by DSS is characterized by impaired epithelial barrier function in the colon. This is manifested by low levels of TJ proteins (occludin, claudin-1, and ZO-1), morphological disruption of intestinal epithelium, and exacerbation of mucosal damage, leading to an increase in the infiltration of inflammatory cytokines into the intestine, forming a vicious cycle [39]. Under normal physiological conditions, MUC2 maintains integrity and lubrication and also protects intestinal mucosal epithelium [40,41,42]. The decrease in MUC2 protein in intestinal tissue suggests that the intestinal barrier is disrupted and the intestinal mucosal defense ability is weakened [43]. Through TEM observation, this study found that *B. tequilensis* YB-2 alleviated the TJ damage and intestinal mucosal injury caused by DSS. Meanwhile, colonic TJ proteins ZO-1, MUC2, claudin-1, and occludin in colitis mice pre-treated with *B. tequilensis* YB-2 significantly increased their expression levels and played a critical part in maintaining the epithelial barriers of the mouse colon. Recent studies also showed that feeding *B. tequilensis* K03 significantly increased the expression of the MUC2 gene and decreased the content of *E.coli* in ileum of broilers [44]. In addition, studies on other *Bacillus* have also found that oral administration of *B. subtilis* can improve colonic epithelial integrity, with increased protein expression of ZO-1 and occludin [45]. Jia et al. [26] found that *B. licheniformis* ZW3 could ameliorate DSS-induced dysfunction of the colonic barrier by enhancing MUC2, ZO-1, and occludin.

The maintenance of the intestinal epithelial barrier and the NF-κB pathway were closely related, and activating the NF-κB pathway causes TJ molecules to be downregulated and abnormally localized, thereby increasing the permeability of the intestinal epithelial barrier [46,47,48]. NF-κB cannot be activated without the upstream TLR4 [49]. TLR4 is an important recognition receptor for pathogen-associated molecules on the cell surface, and when activated, it leads to enhance downstream NF-κB activation, ultimately leading to the synthesis and release of a large number of pro-inflammatory cytokines, with subsequent injury to the intestinal epithelial barrier [50,51]. The TLR4/NF-κB signaling pathway has close correlation with the colitis severity, which has been well confirmed in the colitis pathogenesis induced by DSS. Li et al. [52] showed that the TLR4/NF-κB pathway is activated in DSS-induced colitis model, and then inflammatory mediators such as TNF-α are induced by NF-κB, causing damage to colonic tissue and barrier functions. This research also found that DSS increased TLR4 protein expression level in the colon tissues of mice and upregulated NF-κB phosphorylation. However, the TLR4 protein expression and NF-κB phosphorylation were suppressed significantly in the colon tissues of mice pre-treated using *B. tequilensis* YB-2, which indicates the effects of *B. tequilensis* YB-2 in reducing inflammatory mediator production and alleviating intestinal barrier damage by suppressing the TLR4/NF-κB pathway. Similar to the above findings, Zhang et al. [33] showed that *Lactobacillus plantarum* can improve the intestinal barrier dysfunctions and inflammatory responses induced by *Clostridium perfringens* by regulating the TLR4/NF-κB pathway and mitochondrial autophagy responses. Hu et al. [53] found that *B. subtilis* H28 suppressed the NF-κB and MAPK pathways to alleviate mouse mastitis induced by *E. coli*, providing a new method for preventing mastitis. Sun et al. [54] orally treated obese rats with *B. subtilis* natto JLCC513 and found that JLCC513 alleviated intestinal barrier dysfunctions induced by a high-fat diet through inhibiting the TLR4/NF-κB/NLRP3 pathway and regulating intestinal flora disorder.

Overall, this study screened out a strain of *B. tequilensis* from pig manure, emphasized its probiotic function in maintaining the intestinal barrier and alleviating intestinal inflammation, and explored its possible probiotic mechanism. However, due to differences in animal models, this study cannot be used as strong evidence for its application in pig production. Therefore, in future studies, we will also add *B. tequilensis* YB-2 to the diet of piglets to carry out relevant experiments to further explore the possibility of its application in pig production.

## 5. Conclusions

**Figure 8:**
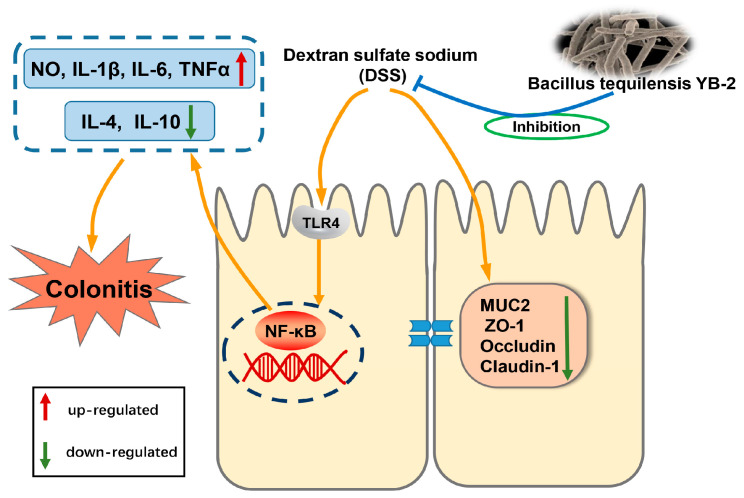
The possible mechanism of *B. tequilensis* YB-2 alleviating colitis in mice.

In this study, we isolated a new probiotic strain, named *B. tequilensis* YB-2, from healthy adult pigs. It has strong acid and bile salt resistance, as well as antimicrobial ability. Through animal experiments, this study found that supplementation of *B. tequilensis* YB-2 in the diet can significantly reduce colitis induced by DSS and alleviate intestinal barrier damage in mice, and its protective mechanism may be achieved by inhibiting the TLR4/NF-κB pathway (Figure 8). These research results provide new strains for developing new feed probiotic preparations, as well as a theoretical basis for applying *B. tequilensis* YB-2 to prevent animal intestinal diseases, which is of great significance in finding green and healthy antibiotic substitutes and promoting the healthy development of animal husbandry.

## Figures and Tables

**Table 1:** The protocol of the animal experiment.

	Control Group	DSS Group	YB-2 Group	DSS + YB-2 Group
	18–22 g Male ICR Mice, n = 18/Group			
1th to 4th week	Sterile saline (i.g)	Sterile saline (i.g)	*B. tequilensis* YB-2 suspension (i.g)	*B. tequilensis* YB-2 suspension (i.g)
5th week	The normal drinking water	3% DSS in the drinking water	The normal drinking water	3% DSS in the drinking water

Note: “i.g” means intragastric administration.

**Table 2:** Sequences of primers used in the study.

Target Gene	Forward (5′-3′)	Reverse (5′-3′)
IL-1β	TCGGCAAAGAAATCAAGATGGC	GTGCAAGTCTCATGAAGTGAGC
IL-6	ACAGAAGGAGTGGCTAAGGA	AGGCATAACGCACTAGGTTT
TNF-α	CGTCGTAGCAAACCACCAAG	TTGAAGAGAACCTGGGAGTAGACA
IL-4	CTTCCAAGGTGCTTCGCATA	GATGAATCCAGGCATCGAAA
IL-10	AATTCCCTGGGTGAGAAGCTGAAG	CTGCTCCACTGCCTTGCTCTTAT
MUC2	AGTCTGCTCGTGAAGTGCC	GGCAAACACAGTCCTTGCAG
ZO-1	CTTCTCTTGCTGGCCCTAAAC	TGGCTTCACTTGAGGTTTCTG
Occludin	CACACTTGCTTGGGACAGAG	TAGCCATAGCCTCCATAGCC
Claudin-1	GGTTATCGGAACTGTGGTAGAA	GTGCTCAGGGAAGATGGTAAG
TLR4	GCCGGAAAGTTATTGTGGTG	ATGGGTTTTAGGCGCAGAGTT
NF-κB	GGGCATGCGTTTCCGTTACA	ATGTGGATGAGGCCGGTGAG
β-actin	GGAGATTACTGCCCTGGCTCCTA	GACTCATCGTACTCCTGCTTGCTG

Note: IL-1β, interleukin-1β; IL-6, interleukin-6; TNF, tumor necrosis factor; IL-4, interleukin-4; IL-10, interleukin-10; MUC2, mucoprotein 2; ZO-1, zonula occludens-1; TLR4, Toll-like receptor 4; NF-κB, nuclear factor kappa-B.

## Data Availability

Data will be made available upon reasonable request.

## Supplementary Materials

The following supporting information can be downloaded at https://www.mdpi.com/article/10.3390/ani14131989/s1, Table S1: *p*-values of significant differences between groups (1); Table S2: *p*-values of significant differences between groups (2); Table S3: *p*-values of significant differences between groups (3); Table S4: *p*-values of significant differences between groups (4); Table S5: *p*-values of significant differences between groups (5).
