# Supplementary material for: Pig-Derived Probiotic Bacillus tequilensis YB-2 Alleviates Intestinal Inflammation and Intestinal Barrier Damage in Colitis Mice by Suppressing the TLR4/NF-κB Signaling Pathway
Source: Animals (Basel). 2024 Jul 5;14(13):1989. doi: 10.3390/ani14131989 (PMC11240761; doi:10.3390/ani14131989)
Supplement: Supplementary file 1 [file animals-14-01989-s001.zip › animals-3050292-supplementary.pdf]

Supplementary Material:

Table S1. ***P*-values of significant differences between groups (1)**

p-Value	Colon Length	Histological scores	Goblet cells numbers
**Control vs. DSS**	0.0007	<0.0001	<0.0001
**Control vs. YB-2**	\	\	\
**Control vs. DSS+YB-2**	\	0.0002	0.0002
**DSS vs. YB-2**	<0.0001	<0.0001	<0.0001
**DSS vs. DSS+YB-2**	\	<0.0001	0.0065
**YB-2 vs. DSS+YB-2**	0.0290	0.0004	<0.0001

Table S2. ***P*-values of significant differences between groups (2)**

p-Value	NO in serum	IL-1β in serum	IL-6 in serum	TNF-α in serum	IL-10 in serum	IL-4 in serum
**Control vs. DSS**	<0.0001	<0.0001	<0.0001	<0.0001	0.0001	<0.0001
**Control vs. YB-2**	\	\	\	\	\	\
**Control vs. DSS+YB-2**	<0.0001	0.0008	0.0409	<0.0001	\	<0.0001
**DSS vs. YB-2**	<0.0001	<0.0001	<0.0001	<0.0001	<0.0001	<0.0001
**DSS vs. DSS+YB-2**	<0.0001	0.0044	0.0175	0.0020	0.0236	<0.0001
**YB-2 vs. DSS+YB-2**	<0.0001	0.0012	0.0214	<0.0001	0.0177	<0.0001

Table S3. ***P*-values of significant differences between groups (3)**

p-Value	NO in colon	IL-1β (qRT-PCR)	IL-6 (qRT-PCR)	TNF-α (qRT-PCR)	IL-10 (qRT-PCR)	IL-4 (qRT-PCR)
**Control vs. DSS**	<0.0001	<0.0001	<0.0001	<0.0001	<0.0001	<0.0001
**Control vs. YB-2**	\	\	\	\	\	\
**Control vs. DSS+YB-2**	0.0076	0.0221	0.0343	0.0130	0.0272	0.0063
**DSS vs. YB-2**	<0.0001	<0.0001	<0.0001	<0.0001	<0.0001	<0.0001
**DSS vs. DSS+YB-2**	<0.0001	<0.0001	<0.0001	0.0003	0.0011	0.0006
**YB-2 vs. DSS+YB-2**	0.0069	0.0219	\	0.0280	0.0523	0.0433

Table S4. P-values of significant differences between groups (4)

p-Value	MUC2 (WB)	ZO-1 (WB)	Occludin (WB)	Claudin (WB)	MUC2 (qRT-PCR)	ZO-1 (qRT-PCR)	Occludin (qRT-PCR)	Claudin-1 (qRT-PCR)
**Control vs. DSS**	<0.0001	<0.0001	<0.0001	<0.0001	<0.0001	0.0002	<0.0001	<0.0001
**Control vs. YB-2**	\	\	\	\	\	\	\	\
**Control vs. DSS+YB-2**	0.0012	<0.0001	0.0061	\	0.0028	0.1663	\	0.0158
**DSS vs. YB-2**	<0.0001	<0.0001	<0.0001	<0.0001	<0.0001	<0.0001	<0.0001	<0.0001
**DSS vs. DSS+YB-2**	0.0380	0.0376	0.0034	<0.0001	0.0112	0.0240	0.0030	0.0231
**YB-2 vs. DSS+YB-2**	0.0184	<0.0001	0.0124	\	0.0012	0.0435	\	0.0027

Table S5. P-values of significant differences between groups (5)

p-Value	TLR4 (WB)	p-NF-κB/-NF-κB (WB)	TLR4 (qRT-PCR)	NF-κB (qRT-PCR)
**Control vs. DSS**	<0.0001	<0.0001	<0.0001	<0.0001
**Control vs. YB-2**	\	\	\	\
**Control vs. DSS+YB-2**	\	0.0384	\	0.0482
**DSS vs. YB-2**	<0.0001	<0.0001	<0.0001	<0.0001
**DSS vs. DSS+YB-2**	0.0003	0.0008	0.0001	0.0395
**YB-2 vs. DSS+YB-2**	0.0325	0.0204	\	0.0298
